# Use of hand-held Doppler ultrasound examination by podiatrists: a reliability study

**DOI:** 10.1186/s13047-015-0097-2

**Published:** 2015-08-12

**Authors:** Peta Ellen Tehan, Vivienne Helaine Chuter

**Affiliations:** School of Health Sciences, Faculty of Health, University of Newcastle, Ourimbah, NSW 2258 Australia

**Keywords:** Doppler, Reliability, Peripheral arterial disease

## Abstract

**Background:**

Hand held Doppler examination is a frequently used non-invasive vascular assessment utilised by podiatrists. Despite this, the reliability of hand-held Doppler has not been thoroughly investigated. Given the importance of Doppler in completing a vascular assessment of the lower limb, it is essential to determine the reliability of the interpretation of this testing method in practicing podiatrists.

**Methods:**

This was a multi-centre inter and intra-rater reliability study. Four podiatrists (the raters) participated in this study, two public and two private practitioners. Three aspects of Doppler use were examined; (i) use of Doppler (i.e., technique and interpretation), (ii) interpretation of Doppler audio sounds, and (iii) interpretation of visual Doppler waveforms (i.e., tracings). Participants meeting current guidelines for vascular screening attended two testing sessions, 1 week apart at either the private practice (*n* = 32), or the public practice (*n* = 31). To assess use of Doppler, the raters evaluated the Doppler waveforms that they collected, rating them as mono-phasic or multi-phasic. To assess Doppler audio sounds and visual Doppler waveforms, raters were required to evaluate 30 audio recordings of Doppler sounds and 30 waveform tracings, respectively, that were previously recorded and chosen at random by the researchers. Cohen’s kappa (κ) statistics were used to calculate inter and intra-rater reliability using SPSS version 19.

**Results:**

Use of Doppler demonstrated the lowest reliability for both pairs of clinicians (inter-rater reliability κ 0.20 to 0.24 and intra-rater reliability κ 0.27 to 0.42). The public podiatrists showed higher reliability in audio interpretation (inter-tester reliability κ 0.61, intra-tester reliability κ 1.00) compared to the private podiatrists (inter-tester reliability κ 0.31, intra-tester reliability κ 0.53). Evaluation of Doppler waveform tracings demonstrated highest reliability, with inter-rater reliability ranging from κ 0.77 to 0.90 and intra-rater reliability from κ 0.81 to 1.00.

**Conclusions:**

There is a need for ongoing education for podiatrists using Doppler in clinical practice, as the reliability for the clinical use of the Doppler was low. This indicates that technique could be an issue. There is also a need to further evaluate if hand-held Doppler equipment, using the examinations that we evaluated, is suitable for use in the contexts examined in this study.

**Electronic supplementary material:**

The online version of this article (doi:10.1186/s13047-015-0097-2) contains supplementary material, which is available to authorized users.

## Background

Peripheral arterial disease (PAD) is associated with cardiovascular morbidity and mortality [1] and the development of lower limb wounds, gangrene and amputation. The condition becomes increasingly prevalent in older age, renal disease and inflammatory arthritis. PAD also occurs earlier, more distally and with more rapid progression in association with diabetes [2, 3]. Early detection is essential to ensure that modifiable risk factors are identified and for the conditions to be appropriately monitored and managed to prevent potentially life-threatening complications.

Regular screening of those at risk of PAD is essential as only 22 % of people with PAD are symptomatic [4]. Current recommendations indicate routine lower limb vascular screening is required for those over the age of 65 years, or over 50 years with diabetes or a history of smoking [5]. Podiatrists are in an ideal position to carry out vascular screening on a regular basis, as people who are older and have diabetes frequently seek podiatric care [6]. With an ageing population and increasing prevalence of diabetes [7], non-invasive vascular screening is becoming increasingly important to prevent lower limb complications related to PAD.

Hand-held Doppler ultrasound examination (Doppler) of pedal arteries is the most frequently used non-invasive vascular assessment modality utilised by podiatrists [8] for diagnosis and ongoing monitoring of PAD. Podiatrists generally use Doppler in two different ways, as part of an ankle brachial index (ABI) or as a standalone test [8]. Doppler examination is a useful method for vascular screening as it has been demonstrated to be effective for detecting and excluding PAD, can be performed at relatively low cost and is non-invasive [9, 10].

In the foot, the dorsalis pedis and posterior tibial arteries are the most frequently examined due to their accessibility [11]. Both audio and visual analyses of Doppler waveforms are performed by clinicians to determine the presence of PAD. In audio analysis non-pathological Doppler waveforms are considered multiphasic, which includes bi-phasic (two) or tri-phasic (three) sounds [12, 13]. In contrast, a monophasic waveform is a single sound that is considered pathological [11], indicating the presence of PAD. In visual analysis of a Doppler tracing, a non-pathological waveform has a distinct shape representing high resistance and diastolic flow reversal, which can be classified as multiphasic (bi or tri-phasic). Pathological waveforms generally have low resistance, slow systolic acceleration and no diastolic flow reversal and are classified as monophasic [11].

The accurate use of Doppler relies upon multiple competencies including the skills involved in accurate application of the device, and concurrent interpretation of both audio and visual data to classify the waveform as normal or pathological. For this type of assessment to be useful for ongoing monitoring of PAD in practice, high reliability of the measurement is required. However, despite its widespread use in the podiatry profession, very little investigation has been completed on the reliability of either clinical measurement or interpretation for this type of assessment.

Currently, evidence of reliability of Doppler use in podiatry practice is isolated to interpretation of audio sound alone, with several studies demonstrating moderate inter-rater reliability [14, 15]. In professions other than podiatry, hand-held Doppler has been shown to have high levels of reliability [10]. A comprehensive assessment of the three elements of Doppler use (clinical application with waveform interpretation and independent audio and visual interpretation of waveforms) is required to determine the clinical efficacy of using this technique for ongoing peripheral vascular monitoring.

The aim of this study was to investigate the inter- and intra-rater reliability of the use of Doppler ultrasound for collection and interpretation of Doppler waveforms by podiatrists in mixed clinical settings. This included: (i) overall use of Doppler to evaluate the pedal pulses (involving conducting the assessment and interpreting audio and visual outputs), (ii) interpretation of Doppler audio sounds presented independently, and (iii) interpretation of visual Doppler waveforms presented independently.

## Methods

This was an inter- and intra-rater reliability study that took place over a period of 6 months (June – November 2013). Ethical approval was obtained from the University of Newcastle and Hunter New England Local Health District Ethics Committees, New South Wales, Australia (Reference number 13/02/20/5.05). All participants signed informed consent prior to being recruited into the study.

### Raters

Four podiatrists (i.e., the raters) with varying levels of clinical experience (1–8 years) who studied at three different tertiary institutions across two states of Australia were invited, and subsequently agreed to participate in this study. The raters were selected to ensure varying levels of experience, training and employment sector were included. Written informed consent was obtained from each participating podiatrist. All raters had previous experience with use of Doppler ultrasound for lower limb vascular assessment and did not receive further instruction on how to perform this task.

### Participants

A convenience sample from the patient populations at each respective clinic were recruited for this study. In accordance with current guidelines for lower extremity vascular screening, eligibility criteria were: people aged over 65 years, or, aged over 50 years with a history of diabetes or smoking, or with exertional leg pain or non-healing wounds [16]. This group was chosen as it is representative of people who may undergo these tests in clinical practice. Exclusion criteria were: contraindications to Doppler testing including active foot or leg ulceration preventing Doppler placement, known allergy to coupling gel and/or an inability to lie supine for more than 20 min.

### Procedure

**Fig. 1:**
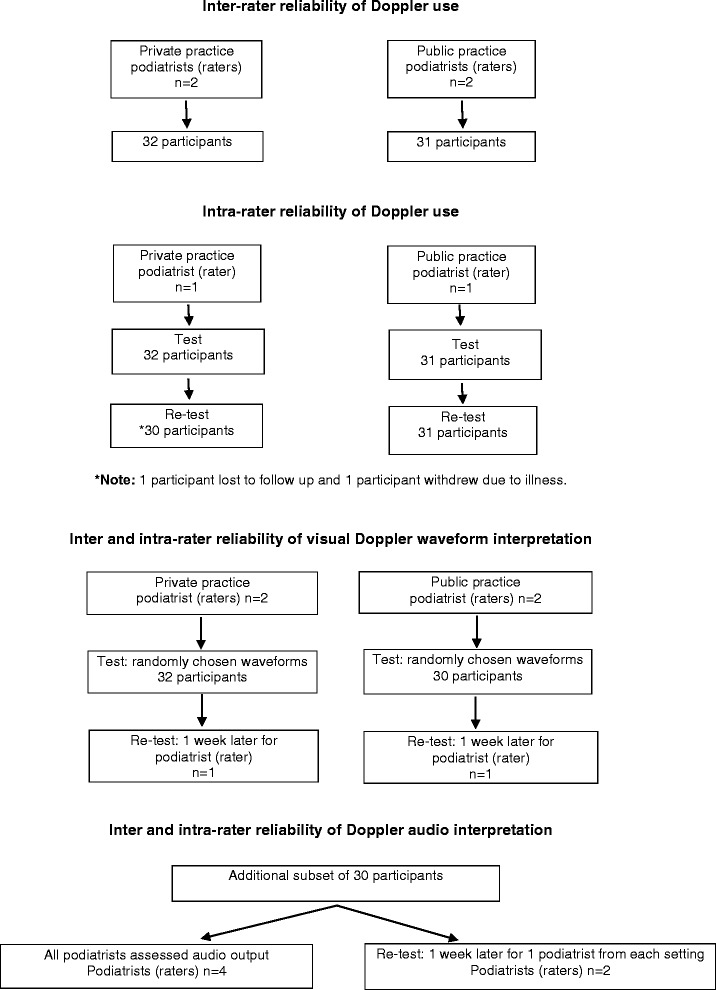
Flow diagrams for the different components of the study

Two testing sites were used, one was a podiatry clinic in a community health centre (public practice) in the Newcastle area (New South Wales, Australia) and one was a private podiatry clinic (private practice) in the same catchment. Participants were assessed at the testing site of the service they attended (Fig. 1). All participants were instructed to avoid exercise, caffeine and smoking for at least 1 h prior to their assessment as these are known to affect vascular assessment [17]. All assessments were undertaken in a quiet, private room. Raters were blinded to both their own and each other’s results at all times. To ensure consistency with data collection, and minimise measurement and interpretation errors [18], a strict data collection protocol was used (Additional file 1).

### Inter- and intra- rater reliability of Doppler use

For this part of the study the inter- and intra-rater reliability of podiatrists performing a Doppler ultrasound assessment of the dorsalis pedis and posterior tibial arteries and the podiatrists ability to interpret their results (i.e., use of the Doppler) was investigated. Participants at each setting were placed in a horizontal supine position and rested for at least 10 min prior to the assessment. To assess inter-rater reliability of clinical use of the Doppler, all podiatrists were required to independently assess dorsalis pedis and posterior tibial arterial flow using a Hadeco Smartdop 45® (Hadeco, Kawasaki) and Aquasonic® ultrasound transmission gel (Parker Laboratories, New Jersey). All testing equipment was new at the beginning of the study. The private practice podiatrists undertook assessment on participants attending the private clinic, and the public sector podiatrists undertook assessments on participants attending the community health podiatry clinic. Based on the audio and visual waveforms produced by their own Doppler assessments, all podiatrists then graded Doppler waveforms as absent, monophasic or multiphasic. All participants returned 1 week later to their original test site, either the public or private practice. Following the same test protocol, each participant had their waveforms obtained and graded again by one of the podiatrists from their previous testing session using the same procedure described previously.

### Inter- and intra-rater reliability of Doppler audio interpretation

To determine the reliability of interpretation of Doppler audio alone, a single researcher (PT), who was not a rater in this study recorded dorsalis pedis and posterior tibial waveforms using the Hadeco Smartdop 45® from a separate, additional subset of 30 eligible participants recruited to the community health centre. Participants were rested in horizontal supine position for a minimum of 10 min prior to assessment. Doppler audio were recorded using a digital Dictaphone held approximately 10 cm from the Doppler speaker. Each set of Doppler audio were recorded for 20 s with the Doppler volume set at high. Either the dorsalis pedis or posterior tibial waveform was then randomly selected for each participant. To determine inter-rater reliability the same selected waveform audio files were then separately played to the four participating podiatrists who evaluated them independently as monophasic or multiphasic. To determine the intra-rater reliability one of the private podiatrists, and one of the public podiatrists repeated the assessment of the same 30 audio files 1 week later, with the order of presentation of the audio files randomised to avoid order error.

### Inter- and intra-rater reliability of visual Doppler waveform interpretation

To isolate reliability of visual interpretation of Doppler waveforms a researcher (PT) who was not a rater in this study, randomly chose 30 printed Doppler waveforms (i.e., tracings) collected by the four raters involved in this study. Each rater was then asked to rate them as monophasic or multiphasic based on the printed waveform. One of the private podiatrists, and one of the public podiatrists repeated the assessment 1 week later using the same set of 30 printed waveforms with the order randomised.

### Data analysis

Inter-rater reliability of (i) waveform interpretation for clinical use of the Doppler, (ii) interpretation of independently collected audio recordings, and (iii) interpretation of independently collected visual wave forms between the two private podiatrists and between the two public podiatrists was calculated by determining the level of agreement between measures using an unweighted Cohen’s kappa (κ) statistic with 95 % confidence intervals. All waveforms were classified as pathological (absent or monophasic) or non-pathological (multiphasic). Intra-rater reliability was calculated in the same manner for one of the public podiatrists and one of the private podiatrists for the three aspects of Doppler use detailed above.

Results were interpreted in accordance with Landis and Koch: ≥0.75 denotes excellent agreement; >0.40 but <0.75 denotes fair to good agreement; and <0.40 denotes poor agreement [19]. All reliability analyses were conducted using SPSS version 19.

## Results

Thirty two participants attended the private practice and 31 participants attended the public practice. Of these, according to the inclusion criteria, 23 (public group) and 15 (private group) were over 50 years of age with diabetes, and 9 (public group) and 15 (private group) were over 65 years of age. No participants had active wounds or exertional leg pain, and only one participant was a current smoker (private group). In the public participant group, there was a larger age range and lower mean age than the private participant group. The public participant group also had higher rates of diabetes than the private participant group. Participant characteristics are listed in Table 1.

**Table 1:** Participant characteristics

	Public participants	Private participants	Audio interpretation
Males n (%)	17 (53)	18 (58)	17 (56)
Females n (%)	15 (47)	13 (42)	13 (44)
Mean age (years)	70.9 (SD 7.1)	72.0 (SD 5.7)	71.6 (SD 6.7)
Age range (years)	57 - 88	61 - 81	55 - 82
DM n (%)	23 (72)	15 (48)	19 (63)
Total N	32	31	30

For Doppler use the public participant group was evaluated by the public practice raters, and private participants were evaluated by private practice raters. For visual Doppler waveform analysis, a sub-set of 30 printed waveforms from both public and private participants were randomly selected and evaluated by all raters. For audio interpretation all raters evaluated the recorded sounds of the sub-group listed above

*SD* standard deviation, *DM* diabetes mellitus

### Inter- and intra-rater reliability of Doppler use

Inter-rater reliability for use of Doppler was poor between the private podiatrists and between public podiatrists for both dorsalis pedis and posterior tibial arteries (Table 2) with 95 % confidence intervals crossing zero. The private podiatrist demonstrated the highest intra-rater reliability for collection and classification of Doppler waveforms for the posterior tibial artery examination (κ: 0.42), which corresponds to fair agreement. Intra-rater reliability was poor for both dorsalis pedis (κ: 0.21) and posterior tibial artery waveforms collected and classified by the public podiatrist (κ: 0.27).

**Table 2:** Reliability results for use of Doppler

	Inter-rater reliability					Intra-rater reliability			
	DP	95 % CI	PT	95 % CI		DP	95 % CI	PT	95 % CI
Private	κ 0.20 (*N* = 32)	−0.09 to 0.49	κ 0.16 (*N* = 32)	−0.11 to 0.43	Private	κ 0.22 (*N* = 30)	−0.31 to 0.53	κ 0.42 (*N* = 30)	0.15 to 0.69
Public	κ 0.17 (*N* = 31)	−0.14 to 0.48	κ 0.24 (*N* = 31)	−0.07 to 0.55	Public	κ 0.21 (*N* = 31)	−0.16 to 0.58	κ 0.27 (*N* = 31)	−0.06 to 0.60

*95 % CI* 95 % confidence intervals, *DP* dorsalis pedis artery, *PT* posterior tibial artery, *Private* private practitioners, *Public* public practitioners

### Inter- and intra-rater reliability of Doppler audio interpretation

Reliability of Doppler audio interpretation was fair for public podiatrists (κ: 0.61) and poor for the private podiatrists (κ: 0.31) (Table 3). Intra-rater reliability of Doppler audio interpretation was excellent for the public podiatrist (κ: 1.00) and fair for the private podiatrist (κ: 0.53).

**Table 3:** Reliability results for Doppler audio interpretation

	Inter-rater reliability	95 % CI		Intra-rater reliability	95 % CI
Private	κ 0.31 (*N* = 30)	−0.08 to 0.70	Private	κ 0.53 (*N* = 30)	0.16 to 0.91
Public	κ 0.61 (*N* = 30)	0.23 to 0.99	Public	κ 1.00 (*N* = 30)	1.00 to 1.00

*95 % CI* 95 % confidence intervals, *Private* private practitioners, *Public* public practitioners

### Inter- and intra-rater reliability of visual Doppler waveform interpretation

The inter-rater reliability of visual Doppler waveform interpretation was excellent for both the private and public podiatrist (κ: 0.90 and κ: 0.77 respectively) (Table 4). Similarly, intra-rater reliability of visual interpretation of the waveforms for both the private podiatrist and public podiatrist were excellent (κ: 1.00 and κ: 0.81 respectively).

**Table 4:** Reliability results for Doppler visual interpretation

	Inter-rater reliability	95 % CI		Intra-rater reliability	95 % CI
Private	κ 0.90 (*N* = 32)	0.71 to 1.09	Private	κ 1.00 (*N* = 32)	1.00 to 1.00
Public	κ 0.77 (*N* = 30)	0.53 to 1.01	Public	κ 0.81 (*N* = 30)	0.57 to 1.05

*95 % CI* 95 % confidence intervals, *Private* private practitioners, *Public* public Practitioners

## Discussion

To the best of the authors’ knowledge this is the first study to examine the reliability of the use of Doppler and waveform interpretation skills in podiatrists. Our results demonstrate that the reliability of Doppler use with classification of waveforms was generally poor. Interpretation of independently collected Doppler audio demonstrated moderate inter-rater reliability and moderate to excellent intra-rater reliability. Finally, visual Doppler waveform interpretation of independently collected waveforms yielded excellent inter-rater and intra-rater reliability in both private and public podiatrists.

These results suggest podiatrists had higher skill level in interpretation of visual waveforms and audio of Doppler waveforms in isolation than when the assessment had to be performed and the visual and audio results interpreted concurrently in a clinical setting. Generally, the 95 % confidence intervals for inter- and intra- rater reliability of the clinical use of Doppler included a negative lower limit. This suggests the range of plausible values for the “true” value of kappa included levels of agreement less than zero, which would be worse than the level of agreement expected from chance alone; that is, if the raters were to guess each rating [20]. The poor levels of agreement between and within clinicians for this aspect of the study may have been related to clinical technique in Doppler use or increased difficulty associated with interpreting visual and audio results simultaneously.

From a clinical perspective Doppler use can be difficult, particularly if patients have issues such as peripheral oedema, if there is fibrosis or adipose tissue present and/or there is anatomical variation in artery location. Such factors affecting reliable performance of the assessment may, therefore, have contributed to poorer reliability seen in this aspect of Doppler use. In addition, the requirement in this present study for clinicians to interpret both visual and audio outputs concurrently to inform their decision on presence or absence of pathology may have resulted in poorer reliability. Higher reliability may have been achieved by reducing the output of the Doppler to one variable, either audio or visual waveform to make the interpretation process more simple. However, as podiatrists are required to do both simultaneously in clinical practice, our results suggest that further training in Doppler use including concurrent interpretation of visual and audio waveforms, is required for this to be an effective component of non-invasive vascular assessment.

Visual Doppler waveform analysis of independently collected waveforms had the most consistently high inter- and intra-rater reliability in this study. As far as we are aware, this is the first study to examine the reliability of visual Doppler waveform analysis in podiatrists. Based on our results, when visual waveform tracings alone were presented to podiatrists in both private and public practices they were able to reliably classify pathological or non-pathological waveforms between themselves and on a test-retest basis. However, interpretation of Doppler audio of waveforms showed much more variable reliability between the two tester groups. Whilst public podiatrists had reasonable inter-rater reliability for interpretation of audio data (κ: 0.61) and perfect intra-rater reliability (κ: 1.00), the private podiatrists had lower inter- and intra- rater reliability (ranging from κ: 0.31 to κ: 0.53).

Previous studies have shown much higher levels of reliability in analysis of audio waveforms in podiatrists [14, 15]. The differences in reliability between private and public sector podiatrists may be due in part, to the differences between the public and private participant (i.e., patient) groups. Although this study did not include any assessment of diagnostic accuracy of the Doppler for PAD, the participant group assessed by the public podiatrists had double the incidence of diabetes. Given increased rates and severity of PAD in this population [21], it is possible that more severe disease was present, which was more easily detected and interpreted resulting in higher reliability.

The low reliability of clinical use of Doppler for peripheral arterial assessment demonstrated in this present study poses significant implications for ongoing patient care. Vascular assessments of patients tend to occur annually and are interpreted relative to previous results. The reliability of assessments is essential for accurate and appropriate management. Given the poor reliability of Doppler use that we found in this study, reliance on this test in isolation is problematic. Our results suggest that, in the small sample of podiatrists we studied, Doppler assessments are of limited use as a tool for ongoing monitoring in clinical practice and, at the very least, it is essential for other objective vascular tests (e.g., Ankle Brachial Index) to be incorporated in the annual screening process. Research has demonstrated that reliability of use and interpretation of Doppler has been achieved in other professions supporting the use of this form of assessment for ongoing monitoring in clinical practice [10, 22]. Although Australia does not currently have any specific guidelines for lower limb vascular assessment in the general population at risk of PAD, the United Kingdom currently use National Institute for health Care Excellence (NICE) guidelines, which recommend documentation and analysis of Doppler waveforms as part of an overall vascular assessment [23]. Our results suggest that further skill development is required specifically for podiatrists to ensure clinical utility of Doppler use within the profession.

The results of this study need to be interpreted in light of several limitations. Firstly, the type of Doppler used may have influenced this study and it is unknown if similar results would be achieved if Doppler ultrasound units from alternative manufacturers had been used or if participating podiatrists had used their regular equipment. However, the style of Doppler used in this study is one commonly used in clinical practice. Secondly, it was assumed that participating podiatrists had previously been trained in Doppler ultrasound assessment, so additional training was not provided. A training session provided prior to the study may have improved reliability, but we avoided this as we wanted results to be an accurate reflection of current skills of practicing clinicians. Nonetheless, raters were given a strict protocol for data collection, which realistically would be expected to improve the reliability of the assessment. Thirdly, clinical experience levels of raters ranged from 1 to 8 years, which may have affected reliability. Although the least experienced podiatrist demonstrated the highest intra-rater reliability for clinical use of Doppler, so this seems unlikely. Finally, despite our best efforts to include podiatrists with a range of experience and undergraduate training from the two main areas of clinical practice (public and private), the clinicians participating in this study may not have been representative of the podiatry profession as a whole. Further investigation in other samples may assist in establishing the true reliability within the podiatry profession generally.

## Conclusions

This study demonstrated that in Australian podiatrists in private and public practice visual Doppler waveform interpretation is the most reliable aspect of Doppler use, followed by Doppler audio interpretation. The poor reliability of the use of Doppler in the small cohort of practitioners in this study suggests that this form of assessment may be of limited use for ongoing monitoring. This finding highlights the need for clinicians to engage in regular and ongoing continuing education in order to improve both collection of Doppler data and interpretation of visual waveforms and audio sounds concurrently. In addition our results suggest that reliance on only qualitative Doppler assessment for ongoing assessment of lower limb arterial status is problematic and that multiple methods of assessing vascular status should be employed.

## Additional file

Additional file 1:
**Clinical use of Doppler reliability study: Testing protocol for podiatrists.** (DOCX 70 kb)
