# Supplementary material for: Use of hand-held Doppler ultrasound examination by podiatrists: a reliability study
Source: J Foot Ankle Res. 2015 Aug 12;8:36. doi: 10.1186/s13047-015-0097-2 (PMC4534124; doi:10.1186/s13047-015-0097-2)
Supplement: Additional file 1: — Clinical use of Doppler reliability study: Testing protocol for podiatrists. (DOCX 70 kb) [file 13047_2015_97_MOESM1_ESM.docx]

Clinical use of Doppler reliability study: Testing protocol for podiatrists

| Participant Name: |  |
| --- | --- |
| Participant DOB: |  |
|  |  |
| Podiatrist Name: |  |
| Date: |  |
|  |  |
| Testing Session: | First/Repeat |
|  |  |
| Please circle which category your participant fits into: |  |
|  |  |
| Over 65  Over 50 with DM  Over 50 and currently smoking  Exertional leg pain  Current wound | |
|  |  |
| Has your participant abstained from caffeine, exercise and smoking in the past hour? Please circle:  Yes/No | |

Ensure the participant is rested in a lying supine position for ten minutes prior to undertaking Doppler measurement.

All measurements are to be taken of the participants RIGHT side.

Please rate the pulse detected with Doppler as either absent, mono-phasic, bi-phasic or tri-phasic in the box below.

Doppler Measurement:

|  | Dorsalis Pedis (DP) | Posterior Tibial (PT) |
| --- | --- | --- |
| RIGHT |  |  |

Please also print and label the waveform as either DP or PT and attach to this sheet for our records.
